# Supplementary material for: Changes in Glial Support of the Hippocampus during the Development of an Alzheimer’s Disease-like Pathology and Their Correction by Mitochondria-Targeted Antioxidant SkQ1
Source: Int J Mol Sci. 2022 Jan 20;23(3):1134. doi: 10.3390/ijms23031134 (PMC8834695; doi:10.3390/ijms23031134)
Supplement: Supplementary file 1 [file ijms-23-01134-s001.zip › Tables S1 S2 S4 S5 S6.pdf]

International Journal of *Molecular Sciences*

MDPI

Table S1. Distributions of the density of GFAP$^+$ astrocytes throughout the regions of the hippocampus in OXYS and Wistar rats (counts per 10000 μm^2).

Brain area	Wistar 3 m.	OXYS 3 m.	Wistar 18 m.	OXYS 18 m.	Wistar+SkQ1	OXYS+SkQ1
Hippocampus	3.97±0.29	3.69±0.41	2.74±0.21^	3.83±0.13*	3.56±0.20#	3.53±0.13
CA1 region	3.86±0.26	3.51±0.36	2.35±0.18^	3.43±0.15*	3.18±0.34#	3.42±0.14
Pyramidal layer of CA1	3.83±0.39	3.94±0.49	2.43±0.28^	4.14±0.25*	3.95±0.67#	3.94±0.47
Molecular layer of CA1	3.90±0.27	3.39±0.34	2.34±0.17^	3.30±0.15*	3.06±0.30#	3.33±0.10
CA3 region	5.24±0.77	5.64±0.86	3.09±0.28^	5.15±0.31*	4.48±0.50#	3.75±0.39#
Pyramidal layer of CA3	5.72±0.71	5.83±0.88	2.96±0.28^	6.86±0.55*	5.60±1.09#	4.27±0.57#
Molecular layer of CA3	5.23±0.89	5.60±0.88	3.17±0.33^	4.62±0.28*	4.18±0.43	3.52±0.35#
Dentate gyrus	3.61±0.32	2.51±0.27*	3.21±0.27	4.01±0.15*^	3.80±0.24	3.67±0.14
Granular layer of DG	2.61±0.33	1.68±0.21*	1.74±0.24^	2.62±0.21*^	2.34±0.36	2.78±0.26
Molecular layer of DG	3.22±0.32	2.12±0.26*	3.20±0.33	3.89±0.17^	3.88±0.27	3.63±0.09
Hilus	6.38±0.64	4.93±0.54	5.30±0.36	6.49±0.48	5.41±0.59	5.17±0.56

*$p < 0.05$ for differences between the strains; ^$p < 0.05$ for differences from previous age; #$p < 0.05$ for SkQ1 effects; m.: months (age).

Table S2. Distributions of the density of vimentin$^+$ astrocytes throughout the regions of the hippocampus in OXYS and Wistar rats (counts per 10000 μm^2).

Brain area	Wistar 3 m.	OXYS 3 m.	Wistar 18 m.	OXYS 18 m.	Wistar+SkQ1	OXYS+SkQ1
Hippocampus	0.28±0.03	0.26±0.04	0.23±0.03	0.38±0.03*^	0.36±0.02#	0.43±0.05
CA1 region	0.28±0.03	0.25±0.04	0.35±0.06	0.55±0.04*^	0.58±0.05#	0.69±0.08
Pyramidal layer of CA1	0.29±0.06	0.26±0.07	0.37±0.08	0.51±0.07^	0.59±0.11	0.47±0.05
Molecular layer of CA1	0.28±0.04	0.24±0.04	0.34±0.06	0.56±0.05*^	0.58±0.04#	0.72±0.09
CA3 region	0.32±0.09	0.35±0.07	0.44±0.04	0.78±0.08*^	0.67±0.06#	0.53±0.04#
Pyramidal layer of CA3	0.38±0.12	0.33±0.08	0.52±0.10	0.95±0.22	0.81±0.15	0.36±0.07
Molecular layer of CA3	0.26±0.07	0.33±0.09	0.43±0.05	0.76±0.06*^	0.61±0.10	0.58±0.07
Dentate gyrus	0.17±0.05	0.17±0.02	0.14±0.02	0.24±0.05	0.04±0.01#	0.04±0.01#
Granular layer of DG	1.05±0.47	0.73±0.23	0.29±0.07	0.31±0.09	0.18±0.04	0.20±0.03
Molecular layer of DG	0.04±0.02	0.02±0.01	0.66±0.08^	0.50±0.07^	0.45±0.02#	0.54±0.08
Hilus	0.03±0.02	0.19±0.13	1.39±0.23^	0.92±0.06^	0.98±0.15	0.73±0.13

*$p < 0.05$ for differences between the strains; ^$p < 0.05$ for differences from previous age; #$p < 0.05$ for SkQ1 effects; m.: months (age).

Table S4. Distributions of the density of resting microglia throughout the regions of the hippocampus in OXYS and Wistar rats (counts per 10000 μm²).

Brain area	Wistar 3 m.	OXYS 3 m.	Wistar 18 m.	OXYS 18 m.	Wistar+SkQ1	OXYS+SkQ1
Hippocampus	0.47±0.07	0.61±0.15	0.62±0.06	0.73±0.06	1.08±0.08#	1.33±0.12#
CA1 region	0.70±0.15	0.34±0.05*	1.04±0.07	1.15±0.05^	1.03±0.09	1.23±0.11
Pyramidal layer of CA1	0.86±0.28	0.28±0.05*	0.98±0.16	0.98±0.11^	0.93±0.09	1.10±0.11
Molecular layer of CA1	0.67±0.12	0.34±0.05*	1.06±0.07^	1.17±0.06^	1.05±0.09	1.26±0.12
CA3 region	0.65±0.18	0.41±0.09	1.30±0.21^	1.37±0.12^	1.12±0.13	1.42±0.11
Pyramidal layer of CA3	0.69±0.24	0.30±0.08	0.92±0.16	1.22±0.20^	1.10±0.15	1.23±0.17
Molecular layer of CA3	0.65±0.17	0.45±0.12	1.42±0.26^	1.44±0.18^	1.12±0.18	1.46±0.13
Dentate gyrus	0.43±0.06	0.40±0.06	1.18±0.10^	1.24±0.08^	1.11±0.10	1.45±0.15
Granular layer of DG	0.51±0.07	0.64±0.12	0.80±0.15	0.96±0.10	0.84±0.16	0.96±0.11
Molecular layer of DG	0.62±0.12	0.54±0.09	1.28±0.13^	1.28±0.07^	1.15±0.09	1.51±0.16
Hilus	0.59±0.13	0.43±0.08	1.06±0.18	1.53±0.22^	1.22±0.16	1.75±0.24

*$p < 0.05$ for differences between the strains; ^$p < 0.05$ for differences from previous age; #$p < 0.05$ for SkQ1 effects; m.: months (age).

Table S5. Distributions of the density of activated microglia throughout the regions of the hippocampus in OXYS and Wistar rats (counts per 10000 μm²).

Brain area	Wistar 3 m.	OXYS 3 m.	Wistar 18 m.	OXYS 18 m.	Wistar+SkQ1	OXYS+SkQ1
Hippocampus	1.08±0.14	1.29±0.07	1.01±0.04	1.12±0.04	0.84±0.06#	0.84±0.04#
CA1 region	1.24±0.18	0.97±0.16	0.82±0.07	0.85±0.06	0.73±0.08	0.71±0.04
Pyramidal layer of CA1	1.44±0.26	0.81±0.15	1.04±0.17	1.20±0.19	0.88±0.14	0.78±0.04
Molecular layer of CA1	1.20±0.16	0.99±0.17	0.78±0.06^	0.81±0.06	0.71±0.07	0.70±0.04
CA3 region	1.84±0.35	1.33±0.19	0.97±0.12	1.28±0.12	0.97±0.07	1.12±0.12
Pyramidal layer of CA3	1.63±0.46	1.03±0.14	1.18±0.22	1.66±0.18^	0.72±0.12	1.37±0.23
Molecular layer of CA3	1.92±0.35	1.45±0.22	0.90±0.15^	1.12±0.13	1.08±0.07	1.03±0.11
Dentate gyrus	1.09±0.21	0.81±0.11	0.91±0.02	0.90±0.07	0.94±0.07	0.96±0.04
Granular layer of DG	1.70±0.48	0.65±0.10	0.97±0.08	0.91±0.10	0.87±0.08	0.77±0.08
Molecular layer of DG	1.04±0.18	0.74±0.13	0.72±0.03	0.63±0.05	0.81±0.06	0.76±0.04
Hilus	3.29±0.69	2.65±0.42	1.64±0.13	2.13±0.17	1.65±0.18	1.97±0.11

^$p < 0.05$ for differences compared to previous age; #$p < 0.05$ for SkQ1 effects; m.: months (age).

Table S6. The number of pyknotic nuclei and their percentage phagocyted by microglia in OXYS and Wistar rats (counts per 10000 μm^2).

	**Wistar 18 m.**	**OXYS 18 m.**	**Wistar+SkQ1**	**OXYS+SkQ1**
Number of pyknotic nuclei per 10000 μm^2	8.90±1.61	6.88±0.92	4.86±0.43$^{\#}$	5.17±1.15
Percentage of pyknotic nuclei phagocyted by microglia	76.39±2.57	83.82±3.49	77.78±3.50	79.89±3.37

$^{\#}p < 0.05$ for SkQ1 effects; m.: months (age).
