# Supplementary material for: Changes in Glial Support of the Hippocampus during the Development of an Alzheimer’s Disease-like Pathology and Their Correction by Mitochondria-Targeted Antioxidant SkQ1
Source: Int J Mol Sci. 2022 Jan 20;23(3):1134. doi: 10.3390/ijms23031134 (PMC8834695; doi:10.3390/ijms23031134)
Supplement: Supplementary file 1 [file ijms-23-01134-s001.zip › Tables S3 S7 Figures S1 S2.pdf]

International Journal of
Molecular Sciences

MDPI

Table S3. DEGs specific for astrocytes in the hippocampus of OXYS and Wistar rats at 5 and 18 months of age.

DEGs upregulated in OXYS rats compared to Wistar rats at 5 months	DEGs downregulated in OXYS rats compared to Wistar rats at 5 months	DEGs upregulated in OXYS rats compared to Wistar rats at 18 months	DEGs downregulated in OXYS rats compared to Wistar rats at 18 months
Itga7	*Ptplb*	*Fosb*	*Bdkrb2*
Slc1a4	*Bdkrb2*	*Tom1l1*	*Cth*
Fosb	*Cth*	*Acsf2*	*Cyp1b1*
Tom1l1	*Cyp1b1*	*Agt*	*Evc*
	Evc	*Cbs*	*Gem*
	Gem	*Chsy1*	*Itih3*
	Itih3	*Fos*	*Alpk1*
		Gfap	*Dclk2*
		Hes1	*Egfr*
		Igfbp5	*Elovl5*
		Lrtm2	*Igsf11*
		Naprt1	*Il33*
		Pamr1	*Kcna2*
		Plagl1	*Megf10*
		Ppap2b	*Mtx3*
		Sept9	*Myo6*
		Slc25a34	*Pdgfrb*
		Tpbg	*Pls1*
			Prodh
			Rgs20
			Scara3
			Slc15a2
			Slc30a10
			Slc7a11
			Slc7a2
			Sparcl1
			Tmc7
			Zfp217
**DEGs upregulated in Wistar rats at 18 compared to 5 months of age**	**DEGs downregulated in Wistar rats at 18 compared to 5 months of age**	**DEGs upregulated in OXYS rats at 18 compared to 5 months of age**	**DEGs downregulated in OXYS rats at 18 compared to 5 months of age**
Abhd1	*Thbs1*	*Abhd1*	*Thbs1*
Adhfe1	*Ncan*	*Adhfe1*	*Ncan*
Aldoc	*Lpp*	*Aldoc*	*Lpp*
Als2cl	*Ltbp1*	*Als2cl*	*Ltbp1*
Ccdc157	*Rasgrp1*	*Ccdc157*	*Rasgrp1*
Col16a1	*Sema6d*	*Col16a1*	*Sema6d*
Echdc2	*Gabrb1*	*Echdc2*	*Gabrb1*
Etv4	*Ank2*	*Etv4*	*Ank2*
Ezr	*Slc1a2*	*Ezr*	*Slc1a2*
F3	*Notch3*	*F3*	*Notch3*
Fgfr3	*Znrf3*	*Fgfr3*	*Znrf3*
Fxyd1	*Lama2*	*Fxyd1*	*Lama2*
Fzd9	*Rab30*	*Fzd9*	*Rab30*

Gli1	*Slc7a11*	*Gli1*	*Slc7a11*
Hes1	*Slc7a2*	*Hes1*	*Slc7a2*
Hmgn3	*Ednrb*	*Hmgn3*	*Ednrb*
Hsd11b1	*Kcna2*	*Hsd11b1*	*Kcna2*
Htra1	*Ptplad1*	*Htra1*	*Ptplad1*
Id1	*Nr4a2*	*Id1*	*Nr4a2*
Id3	*Egfr*	*Id3*	*Egfr*
Itih3	*Grm5*	*Itih3*	*Grm5*
Klf15	*Gabbr2*	*Klf15*	*Gabbr2*
Mt3	*Col4a5*	*Mt3*	*Col4a5*
Mtmr11	*Slc4a4*	*Mtmr11*	*Slc4a4*
Naaa	*Dclk1*	*Naaa*	*Dclk1*
Naprt1	*Sorl1*	*Naprt1*	*Sorl1*
Ndrg2	*Ptprz1*	*Ndrg2*	*Ptprz1*
Paqr6	*Agl*	*Paqr6*	*Agl*
Pla2g3	*Igfbp5*	*Pla2g3*	*Igfbp5*
Prdx6	*Gja1*	*Prdx6*	*Gja1*
Prodh	*Prrx1*	*Prodh*	*Prrx1*
Rapgef3	*Shc3*	*Rapgef3*	*Shc3*
Rnf215	*Bmpr1a*	*Rnf215*	*Bmpr1a*
Slc13a5	*Cyp1b1*	*Slc13a5*	*Cyp1b1*
Slc25a42	*Dag1*	*Slc25a42*	*Dag1*
Slc26a6	*Nr4a3*	*Slc26a6*	*Nr4a3*
Slc7a10	*Atp2b4*	*Slc7a10*	*Atp2b4*
Upp1	*Trps1*	*Upp1*	*Trps1*
C4b	*Sash1*	*Acsbg1*	*Sash1*
Cntfr	*Tmem229a*	*Agt*	*Tmem229a*
Cyp4f17	*Tmem56*	*Btbd17*	*Tmem56*
Daam2	*Nr3c2*	*Camk2g*	*Nr3c2*
Dclk2	*Nedd9*	*Cbr3*	*Nedd9*
Fbrsl1	*Dtna*	*Cbs*	*Dtna*
Gjb6	*Myo6*	*Ccdc37*	*Myo6*
Itga7	*Fat1*	*Cldn10*	*Fat1*
Leng8	*Npas3*	*Clu*	*Npas3*
Mgst1	*Arhgap26*	*Ephx2*	*Arhgap26*
Mro	*Itga6*	*Fgfbp3*	*Itga6*
Muc1	*Phka1*	*Fgfr2*	*Phka1*
Pdk4	*Abca1*	*Gabbr1*	*Abca1*
Ppp1r3g	*Ptar1*	*Gadd45g*	*Ptar1*
Slc9a3r1	*Vcl*	*Gfap*	*Vcl*
Tnfaip8	*Sept11*	*Hhipl1*	*Sept11*
Vegfa	*Ntrk3*	*Jun*	*Ntrk3*
	Amot	*Lhx2*	*Amot*
	Smo	*Lxn*	*Smo*
	Rorb	*Mfge8*	*Rorb*
	Elovl5	*Mlc1*	*Elovl5*
	Elovl2	*Nr2e1*	*Elovl2*
	Fmn2	*Nrarp*	*Fmn2*
	Plagl1	*Ntsr2*	*Plagl1*
	Soat1	*Paqr7*	*Soat1*

Ppp1r12a	*Pdpn*	*Ppp1r12a*
Eea1	*Sept9*	*Eea1*
Slc6a11	*Slc25a18*	*Slc6a11*
Bai3	*Slc25a34*	*Bai3*
Dgkb	*Tmem51*	*Dgkb*
Bcl2	*Tspan7*	*Bcl2*
Mboat2	*Tst*	*Mboat2*
Arhgap5	*Ttc14*	*Arhgap5*
Ntrk2	*Wsb1*	*Ntrk2*
Smc4		*Smc4*
Pou3f2		*Pou3f2*
Ccdc80		*Ccdc80*
Slitrk2		*Slitrk2*
Bmpr1b		*Bmpr1b*
Enah		*Enah*
Son		*Son*
Drp2		*Drp2*
Lrrk2		*Lrrk2*
Gli3		*Gli3*
Grm3		*Grm3*
Spry4		*Spry4*
Cd44		*Cd44*
Adcy2		*Adcy2*
Limch1		*Limch1*
Zdhhc21		*Zdhhc21*
Adcyap1r1		*Adcyap1r1*
Trib2		*Trib2*
Rnf24		*Rnf24*
St6galnac5		*St6galnac5*
Kcnn3		*Kcnn3*
Arhgef26		*Arhgef26*
Nhsl1		*Nhsl1*
Slc14a1		*Slc14a1*
Ehbp1		*Ehbp1*
Dock7		*Dock7*
Cd38		*Gcnt4*
Chsy1		*Sfrp1*
Pou3f4		*Tmc7*
Cbx5		*Tmem47*
Sox9		*Slc30a10*
Hapln1		*Aqp4*
Il17rd		*Hgf*
Abcd2		*Alpk1*
Rnf180		*Trpm3*
Sfxn5		*Mfap3l*
Ppp1r3c		*Fgd6*
Slc6a1		*Plce1*
Dab1		*Emp2*
Palld		*Pitpnc1*
		Pdzrn3

			Slc1a4
			Cyp7b1
			Ckap5
			Pdgfrb
			Nrcam
			Car8
			Kcnj10
			Rb1
			Lonrf3
			Tgfb2
			Ptpn13
			Igsf11
			Prkd1
			Tril
			Alcam
			Prdm16
			Cachd1
			Rgs20
			Dpf3
			Timp3
			Arap2
			Ptch1
			Rhoq
			Axl
			Megf10
			Stk38l
			Cpeb4
			Rfx4
			Lrp4
			Olfml1
			Kalrn
			Pou3f3
			Psd2

**SkQ1 prevents upregulation of these DEGs from 5 to 18 months in OXYS rats**	**SkQ1 prevents downregulation of these DEGs from 5 to 18 months in OXYS rats**	**SkQ1 normalizes these DEGs upregulated in OXYS rats compared to Wistar rats at 18 months of age**	**SkQ1 normalizes these DEGs downregulated in OXYS rats compared to Wistar rats at 18 months of age**
Jun	*Alpk1*	*Chsy1*	*Alpk1*
Sept9	*Tmc7*	*Hes1*	*Bdkrb2*
Hhipl1	*Tmem47*	*Plagl1*	*Egfr*
Paqr7	*Slc30a10*	*Ppap2b*	*Elovl5*
	Pdgfrb	*Sept9*	*Evc*
	Rgs20	*Tpbg*	*Gem*
	Megf10		*Il33*
			Kcna2
			Megf10
			Myo6
			Pdgfrb
			Pls1

	Rgs20
	Scara3
	Slc15a2
	Slc30a10
	Slc7a11
	Slc7a2
	Tmc7

Figure S1. Correlations between densities of vimentin⁺ astrocytes (considered "reactive") and CD68⁺ microglia (regarded as "activated") in hippocampal regions of OXYS and Wistar rats at 18 months of age as well as after SkQ1 treatment.

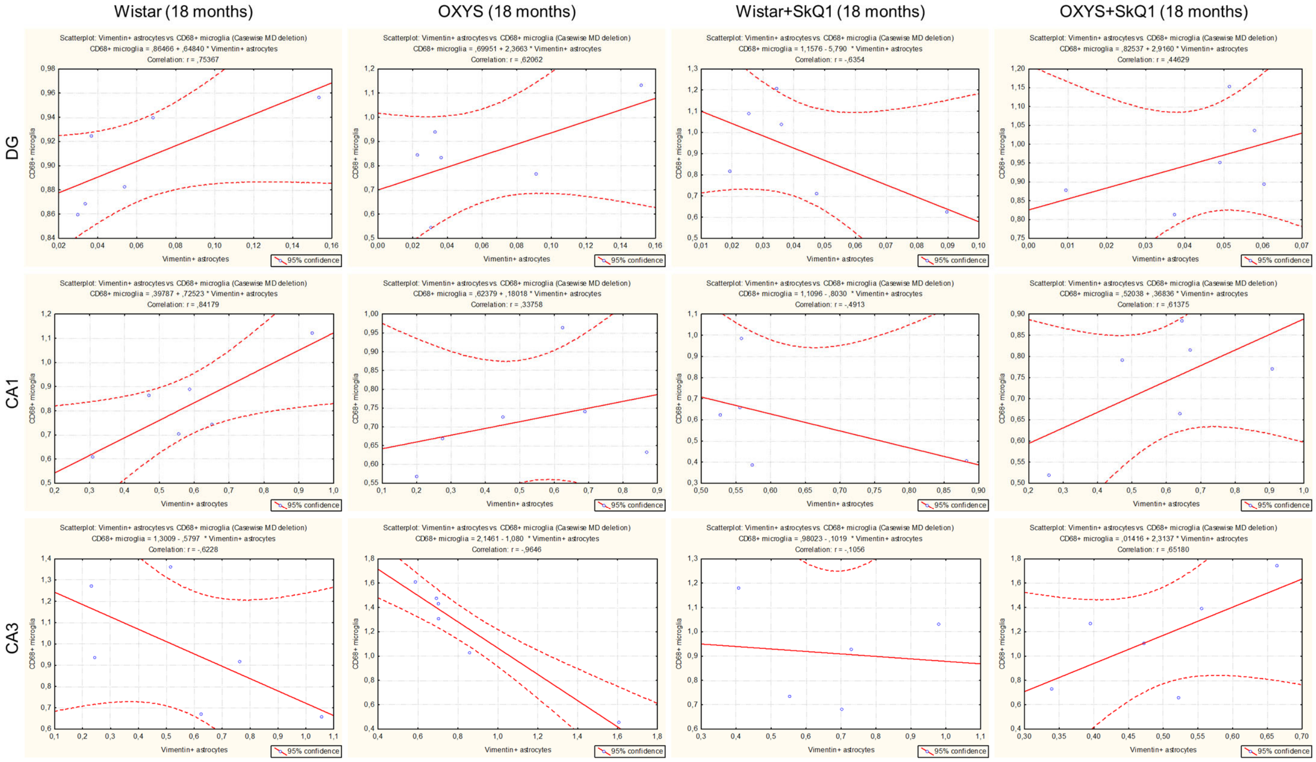

Figure S2. Representative immunohistochemical images of a microglial cell (indicated by an arrow) that is phagocytosing a pyknotic nucleus (indicated by a dashed arrow) in the molecular layer of the CA1 hippocampal area of a Wistar rat at 3 months of age. Iba1 is stained green; DAPI (blue) indicates cell nuclei. The scale bar is 20 μm.

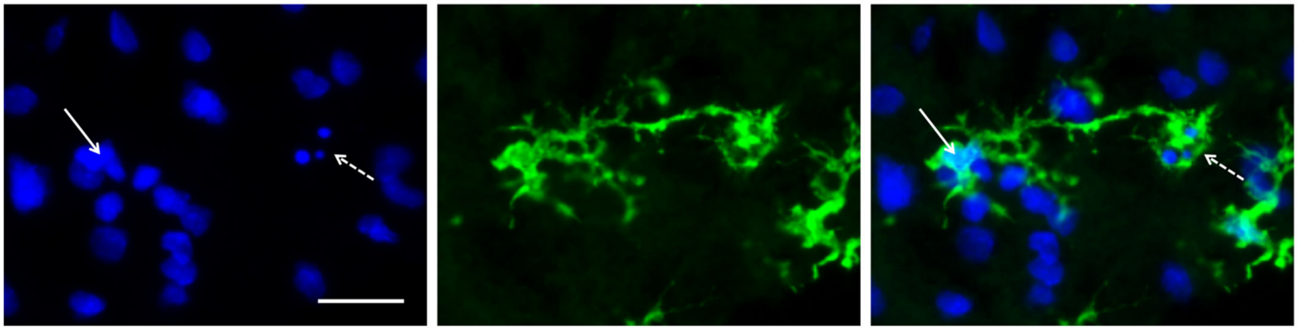

Table S7. DEGs specific for microglia in the hippocampus of OXYS and Wistar rats at 5 and 18 months of age.

DEGs upregulated in OXYS rats compared to Wistar rats at 5 months	DEGs downregulated in OXYS rats compared to Wistar rats at 5 months	DEGs upregulated in OXYS rats compared to Wistar rats at 18 months	DEGs downregulated in OXYS rats compared to Wistar rats at 18 months
Aldh16a1	*Cd48*	*Cebpb*	*Abca9*
Map3k14	*Cyp4v3*	*Efhd2*	*Adap2*
		Egr2	*Apobec1*
		Junb	*Cd48*
		Map3k14	*Cdk6*
		P2ry2	*Ctss*
		Paox	*Cyp4v3*
		Plekho1	*Hk2*
		Ptgs1	*Mlxipl*
		Rgs14	*Nhlrc3*
		Syngr1	*P2ry12*
		Tmem206	*Pik3cg*
		Tor3a	*S100a9*
		Vps18	*Scpep1*
			Skap2
			Slc11a1
			Slc2a5
			Spp1
			Tlr13
			Ubc
**DEGs upregulated in Wistar rats at 18 compared to 5 months of age**	**DEGs downregulated in Wistar rats at 18 compared to 5 months of age**	**DEGs upregulated in OXYS rats at 18 compared to 5 months of age**	**DEGs downregulated in OXYS rats at 18 compared to 5 months of age**
Abhd12	*Atp8a2*	*Abhd12*	*Atp8a2*
Abi3	*Dtx4*	*Abi3*	*Dtx4*
Acads	*Edem1*	*Acads*	*Edem1*
Aif1	*Gpr165*	*Aif1*	*Gpr165*
Aldh16a1	*Hivep3*	*Aldh16a1*	*Hivep3*
Alox5ap	*Kctd12*	*Alox5ap*	*Kctd12*
Arl11	*Pdxk*	*Arl11*	*Pdxk*
Armc5	*Plxdc2*	*Armc5*	*Plxdc2*
Arrb2	*Sdc4*	*Arrb2*	*Sdc4*
Asb2	*Sh3pxd2a*	*Asb2*	*Sh3pxd2a*
Blnk	*Sirpa*	*Blnk*	*Sirpa*
C1qa	*Slc7a8*	*C1qa*	*Slc7a8*
C1qb	*Tbc1d2b*	*C1qb*	*Tbc1d2b*
C1qc	*Tgfbr1*	*C1qc*	*Tgfbr1*
Cables1	*B4galt5*	*Cables1*	*Abca9*
Cd300a	*Egr1*	*Cd300a*	*Fam46c*
Cd33	*Egr2*	*Cd33*	*Gpr137b*
Cd37	*Egr3*	*Cd37*	*Hk2*
Cd68	*Gcnt1*	*Cd68*	*Lcp1*
Cebpa	*Lasp1*	*Cebpa*	*Orai2*
Cebpb	*Syngr1*	*Cebpb*	*Pik3cg*

Clic1	*Tatdn2*	*Clic1*	*Plagl2*
Col27a1		*Col27a1*	*Plekho2*
Comtd1		*Comtd1*	*Slc11a1*
Cotl1		*Cotl1*	*Ubash3b*
Crybb1		*Crybb1*	*Ubc*
Cst3		*Cst3*	
Ctsa		*Ctsa*	
Ctsb		*Ctsb*	
Ctsd		*Ctsd*	
Ctsf		*Ctsf*	
Ctss		*Ctss*	
Ctsz		*Ctsz*	
Cyba		*Cyba*	
Cyth4		*Cyth4*	
Dcxr		*Dcxr*	
Dusp5		*Dusp5*	
Erp29		*Erp29*	
Fcer1g		*Fcer1g*	
Fermt3		*Fermt3*	
Fes		*Fes*	
Fgd2		*Fgd2*	
Gadd45b		*Gadd45b*	
Galns		*Galns*	
Gna15		*Gna15*	
Gpr183		*Gpr183*	
Gpr34		*Gpr34*	
Gpr84		*Gpr84*	
Gpx1		*Gpx1*	
Hcls1		*Hcls1*	
Herpud1		*Herpud1*	
Hexa		*Hexa*	
Hexb		*Hexb*	
Hmga1		*Hmga1*	
Hmha1		*Hmha1*	
Ier2		*Ier2*	
Ier5		*Ier5*	
Ifi30		*Ifi30*	
Ifngr2		*Ifngr2*	
Irf1		*Irf1*	
Irf8		*Irf8*	
Itgb2		*Itgb2*	
Junb		*Junb*	
Khk		*Khk*	
Klf2		*Klf2*	
Klk8		*Klk8*	
Laptm5		*Laptm5*	
Lfng		*Lfng*	
Lgmn		*Lgmn*	

Lrch4	*Lrch4*
Ltc4s	*Ltc4s*
Lyl1	*Lyl1*
Man2b1	*Man2b1*
Matk	*Matk*
Mcl1	*Mcl1*
Mknk1	*Mknk1*
Nab2	*Nab2*
Naglu	*Naglu*
Nagpa	*Nagpa*
Ncf4	*Ncf4*
Nfkb1	*Nfkb1*
Nfkb2	*Nfkb2*
Nfkbid	*Nfkbid*
Nhp2	*Nhp2*
Ntpcr	*Ntpcr*
Pdgfb	*Pdgfb*
Pdlim4	*Pdlim4*
Pkn1	*Pkn1*
Pld4	*Pld4*
Plek	*Plek*
Pnp	*Pnp*
Pnpla2	*Pnpla2*
Pnpo	*Pnpo*
Ppp1r15a	*Ppp1r15a*
Psmb10	*Psmb10*
Psmb8	*Psmb8*
Ptpn6	*Ptpn6*
Rgs10	*Rgs10*
Rhbdf2	*Rhbdf2*
Rilpl1	*Rilpl1*
Rilpl2	*Rilpl2*
Sap30	*Sap30*
Sat1	*Sat1*
Sbno2	*Sbno2*
Sh3bp1	*Sh3bp1*
Sipa1	*Sipa1*
Skap2	*Skap2*
Snx20	*Snx20*
Sord	*Sord*
Tbc1d10a	*Tbc1d10a*
Tcirg1	*Tcirg1*
Tifab	*Tifab*
Tmc6	*Tmc6*
Tmem120a	*Tmem120a*
Tmem206	*Tmem206*
Tpst2	*Tpst2*
Traf3ip3	*Traf3ip3*

Trem2	*Trem2*
Tspo	*Tspo*
Tyrobp	*Tyrobp*
Uap1l1	*Uap1l1*
Unc13d	*Unc13d*
Unc93b1	*Unc93b1*
Zc3h12a	*Zc3h12a*
Apobec1	*Arhgap22*
Apoc1	*Arhgap25*
Arap1	*Camk1*
Arhgap27	*Ccdc86*
Arhgap4	*Cib1*
Arhgap9	*Cln3*
B2m	*Coro1a*
Casp4	*Dok3*
Cd163	*Ebi3*
Cd53	*Edem2*
Cd74	*Fam105b*
Ch25h	*Fam110a*
Clec10a	*Gpsm3*
Clec4a3	*Jdp2*
Cmtm7	*Lat2*
Csf1r	*Map3k14*
Csf3r	*Myc*
Cxcl16	*Myo1f*
Dennd1c	*Nbeal2*
Dok1	*Nfkbib*
Dusp1	*Oaf*
Eif4ebp1	*Orai1*
Emr1	*Plekho1*
Eno3	*Ppcdc*
F11r	*Psd4*
Fcgr2b	*Rnase4*
Fhod1	*Rogdi*
Grn	*Rtn4rl1*
Hck	*Sall1*
Hfe	*Selplg*
Hmox1	*Snta1*
Icam1	*Sun2*
Ier3	*Tln1*
Ifngr1	*Tnfrsf12a*
Itgam	*Twf2*
Leprel1	
Lgals3	
Lgals3bp	
Ly86	
Man2b2	
Map3k8	

Mlxipl			
Ncf1			
Nfkbia			
Nfkbiz			
P2ry12			
P2ry6			
Phf15			
Pik3cd			
Plcg2			
Plod3			
Prkcd			
Psmb9			
Ptpn18			
Pxn			
Rac2			
Ripk3			
S100a9			
Scpep1			
Siglec5			
Slc29a1			
Slc29a3			
Slc2a5			
Socs3			
Sp110			
Spp1			
Stxbp2			
Tcn2			
Tlr6			
Tmem173			
Tnfaip8l2			
Tnfrsf11a			
Tnfrsf1a			
Trpm2			
Trpm4			
Vamp8			
Vav1			
Was			
Zfp36			

**SkQ1 prevents upregulation of these DEGs from 5 to 18 months in OXYS rats**	**SkQ1 prevents downregulation of these DEGs from 5 to 18 months in OXYS rats**	**SkQ1 normalizes these DEGs upregulated in OXYS rats compared to Wistar rats at 18 months of age**	**SkQ1 normalizes these DEGs downregulated in OXYS rats compared to Wistar rats at 18 months of age**
Arhgap22	*Hk2*	*Cebpb*	*Hk2*
Dok3	*Pik3cg*	*Egr2*	*Pik3cg*
Fam110a	*Slc11a1*	*Plekho1*	*Slc11a1*
Plekho1	*Ubc*	*Rgs14*	*Ubc*
Rogdi		*Tmem206*	

Rtn4rl1	*Tor3a*
Snta1	
